# SPARC Promotes Aerobic Glycolysis and 5‐Fluorouracil Resistance in Colorectal Cancer Through the STAT3/HK2 Axis

**DOI:** 10.1002/cam4.70972

**Published:** 2025-05-25

**Authors:** Jingrong Xiang, Huan Zhang, Kanger Shen, Jie Feng, Kexi Yang, Tongguo Shi, Qinhua Xi

**Affiliations:** ^1^ Jiangsu Institute of Clinical Immunology The First Affiliated Hospital of Soochow University Suzhou China; ^2^ Department of Gastroenterology The First Affiliated Hospital of Soochow University Suzhou China; ^3^ Jiangsu Key Laboratory of Clinical Immunology Soochow University Suzhou China

**Keywords:** 5‐FU, aerobic glycolysis, CRC, HK2, SPARC

## Abstract

**Background:**

Chemotherapy has been used extensively in the clinic to treat colorectal cancer (CRC). Nevertheless, cancer cells usually develop chemoresistance under chemotherapy stress, leading to treatment failure. At present, the mechanism of chemoresistance in patients with CRC is not fully understood.

**Methods:**

Firstly, Secreted protein acidic and rich in cysteine (SPARC) expression and prognosis in CRC clinical samples were investigated using tissue microarray (TMAs) and GEPIA databases. Subsequently in vitro, SPARC knockdown or overexpression was used to explore the role of SPARC in 5‐fluorouracil (5‐FU) resistance in CRC cell lines. Western blot or RT‐qPCR was used to analyze the downstream molecules and pathways regulated by SPARC. The contents of glucose and lactic acid were determined by Elisa. In vivo a xenograft tumor model was constructed to verify the function of SPARC in 5‐FU chemoresistance.

**Results:**

This study revealed a correlation between 5‐FU resistance in CRC and the expression of SPARC. The elevated SPARC expression in CRC tissues was linked to a poor prognosis for CRC patients. SPARC knockdown in CRC cells significantly suppressed aerobic glycolysis and 5‐FU resistance, whereas SPARC overexpression had cancer‐promoting effects. Additionally, SPARC increased 5‐FU resistance through the Signal transducer and activator of transcription 3 (STAT3)/Hexokinase‐2 (HK2) pathway. The impact of SPARC on 5‐FU resistance was eliminated both in vitro and in vivo by blocking HK2 or STAT3 signaling.

**Conclusion:**

Our results confirmed that SPARC affects the chemoresistance of CRC to 5‐FU through the STAT3/HK2 axis and is one of the indispensable factors affecting the development of 5‐FU resistance in CRC.

## 1 Introduction

CRC is the second most common cause of cancer‐related death globally and has one of the highest morbidity rates among cancers [1]. Approximately 20% of patients present with metastatic CRC as their initial clinical presentation. In addition, up to 50% of patients with localized disease will eventually develop metastases. Appropriate clinical management of these patients remains a challenging medical problem [2]. On the other hand, neoadjuvant chemotherapy should be considered for all high‐risk patients who are in stages II or above [3]. In clinical settings, chemotherapy has demonstrated a strong anticancer effect [4]. Currently, the standard chemotherapy drugs for CRC patients are oxaliplatin (L‐OHP) and 5‐FU. The treatment regimen typically lasts for either 3 or 6 months, which has been shown to improve patient survival rates [5]. However, medication resistance poses a risk for chemotherapy failure in more than 90% of patients with advanced CRC.

SPARC is also referred to as osteonectin or BM‐40 [6]. The 303‐amino acid protein that the SPARC gene encodes is located on chromosome 5q31‐q33; however, 17 amino acids are eliminated before the protein is secreted [6]. Under physiological conditions, SPARC expression is confined to remodelled tissues such as the gut and bone [7]. However, the expression of SPARC is markedly elevated in a variety of pathological conditions, such as inflammation [8], tumours [9], obesity [10], and diabetes, suggesting that it is associated with the development of a variety of diseases. Increasing evidence has shown that SPARC is expressed at elevated levels in a variety of cancers, such as mesothelioma [11], haematological malignancies [12], osteosarcoma [13], and gastric carcinomas [14], and this phenotype is associated with a poor prognosis. Moreover, SPARC has been demonstrated to be associated with multiple biological functions, including chemoresistance [15, 16]. Ma et al. noted that the recombinant SPARC protein promotes chemosensitivity to 5‐FU in gastric cancer cells by regulating epithelial–mesenchymal transition and cell apoptosis [15]. By activating caspase 8, a SPARC N‐terminal peptide may improve the apoptotic cascade and resensitize chemotherapy‐resistant human colon, breast, and pancreatic cancer cells to treatment [17]. The possible function and mechanism of SPARC in CRC chemoresistance are still unknown.

The Warburg effect, first identified by Otto Warburg in 1924, is a unique occurrence in which tumour cells continue to select, under aerobic conditions, the relatively inefficient ATP‐producing mechanism of glycolysis as their source of energy. It is thought to be a key characteristic of malignancies and indicates a change in the way in which tumour cells use glucose from oxidative phosphorylation to glycolysis. This change in metabolism not only increases tumour malignant potential by making it easier for it to generate energy and associated products but also may help tumour cells survive and grow in other ways by facilitating immune escape, changing the environment around the tumour, and promoting chemoresistance. According to a study by Lin et al., CRC cells proliferate and become more chemoresistant as a result of POU domain class 2 transcription factor 1 (POU2F1)‐mediated promotion of glycolysis and pentose phosphate pathway activity [18]. Furthermore, through metabolic reprogramming, N6‐methyladenosine‐mediated LDHA production may cause CRC cells to develop 5‐FU resistance [19]. Thus, targeting or regulating glycolysis is considered to be one of the most effective strategies for overcoming chemoresistance in malignancies, including CRC.

In this study, we aimed to explore the roles of SPARC in aerobic glycolysis and 5‐FU resistance in CRC.

## 2 Materials and Methods

### 2.1 Clinical Samples and Immunohistochemistry (IHC) Analysis

Clinical samples were analyzed using CRC TMAs (# HColA180Su21) purchased from Shanghai Outdo Biotech Co. Ltd. (Shanghai, China), which contained 92 CRC tissue samples and 86 paraneoplastic tissue samples. The TMAs and in vivo mouse tumor tissues were dehydrated, embedded, sectioned, blocked, incubated with the corresponding primary antibody at 4°C overnight, and incubated with the corresponding secondary antibody at 37°C for 30 min; then, DAB color development, hematoxylin restaining, dehydration, transparency, and sealing steps were performed. The samples were photographed under an inverted microscope (Nikon, Tokyo, Japan) at different magnifications. The scores were based on the intensity of staining and the percentage of positive cells, and the product of the two scores was the final score. Two experienced pathologists blinded to the sample performed scoring. Additional patient characteristics including sex, age, and TNM staging were provided in Table S3. The relevant primary and secondary antibodies are shown in Table S1.

### 2.2 Bioinformatics Analysis

The expression and survival curves of SPARC in CRC patients were also obtained from the GEPIA database.

### 2.3 Cell Culture

RKO and HCT116 human CRC cells were acquired from the American Type Culture Collection (ATCC; Manassas, VA, USA). HCT116 cells were grown in RMPI‐1640 medium (EallBio, #03.4007‐C), whereas RKO cells were grown in DMEM (EallBio, Beijing, China, #03.100‐2C). 10% FBS (EallBio, #3. U16001DC) and 1% penicillin‐streptomycin (NCM Biotech, Suzhou, China, #C100C‐5) were added to the medium. HCT116 and RKO cells were cultivated at 37°C in a humidified incubator with 5% CO_2_. None of these cells were contaminated with mycoplasma.

### 2.4 Western Blot Analysis

The collected cells were lysed via RIPA lysis buffer (Beyotime, Shanghai, China #P0013), and protein samples were obtained via centrifugation at 12000 rpm for 20 min. The protein concentration was determined according to the instructions of the BCA protein assay kit (Beyotime, #P0011). 30 μg of protein samples were electrophoretically separated via 10% SDS–PAGE (NCM Biotech, #P2012), and then, the proteins on the gel were transferred onto a 0.45 μm PDVF membrane (GE Healthcare Life Sciences, Germany, #10600023). The PDVF membrane was blocked with a 5% BSA solution (Fcmacs, Nanjing, China, #FMS‐WB021) for 1.5 h. Following blocking, TBST (1× TBS, 0.1% Tween 20) was used three times to wash the membrane for 10 min each time. After that, the corresponding primary antibody was incubated with the PDVF membrane overnight at 4°C. The following day, the membrane was washed with TBST three times before being incubated with the secondary antibody for 1 h at room temperature (RT). The visualization of immunoreactions was conducted using enhanced chemiluminescence (ECL) reagents (NCM Biotech, #10,100) and a ChemiDoc MP Imaging System (Bio‐Rad, CA, USA). The relevant primary and secondary antibodies are shown in Table S1.

### 2.5 Cell Transfection and Lentivirus Infection

A commercial HK2 siRNA (5′‐GCAGAAGGUUGACCAGUAUTT‐3′) was purchased from Shanghai GenePharma Co. Ltd. (Shanghai, China). HK2 siRNA was transfected at approximately 30% cell density according to the instructions of the Lipo8000 Transfection Reagent (Beyotime, #C0533).

Lentivirus vectors carrying human SPARC cDNA (OE‐SPARC) and the corresponding negative control (NC) were purchased from Shanghai GenePharma Co. Ltd. Additionally, lentiviruses containing a short hairpin RNA (shRNA) targeting human SPARC and its corresponding negative control (sh‐NC) were also obtained from the same company. For lentiviral infection, cells were transfected with lentivirus (MOI: 20) at a density of approximately 30% according to the product instructions. The medium was changed to normal medium 24 h after transfection, and the cells were screened with puromycin 72 h later.

### 2.6 Colony Formation Assay

A total of 1000 cells were spread in a 12‐well plate and cultured in a humidified incubator, and the medium was changed every 3 days for 14 consecutive days. Then, the medium was aspirated, the cells were fixed with 4% paraformaldehyde for 15 min, and stained with crystal violet solution (Beyotime, #C0121). Images were taken via an inverted microscope.

### 2.7 CCK‐8 Assay

A total of 1000 cells were spread in a 96‐well plate and incubated for 4 h before 10 μL of CCK‐8 reagent (NCM Biotech, #C6005) was added to each well. The absorbance was measured at a wavelength of 450 nm via a microplate reader after incubation for 2 h in an incubator.

### 2.8 Apoptosis Assay

The PE Annexin V Apoptosis Detection Kit I (BD Biosciences, NJ, USA, #559763) was used to calculate the cell apoptosis rate. After the cells were collected in flow tubes, they were resuspended in 100 μL of 1× binding buffer, and each tube was then filled with 5 μL of Annexin V and 7‐AAD. Flow cytometry was used to measure the apoptosis rate following a 20‐min dark incubation period at room temperature. Cells that were either Annexin V+/7‐AAD‐ or Annexin V+/7‐AAD+ were classified as apoptotic cells.

### 2.9 Glucose Consumption and Lactate Production Assay

Glucose consumption and lactate production were examined via a glucose assay kit (Robio, Shanghai, #361510) and a lactate assay kit (Jian Cheng, Nanjing, #A019‐3‐1). In brief, the cell culture supernatant was collected for related experiments according to the instructions of the kit, and the absorbance was measured at a wavelength of 450 nm using a microplate reader and used for calculations.

### 2.10 Total RNA Isolation and Real‐Time Quantitative Polymerase Chain Reaction (RT–qPCR)

Cellular RNA was extracted with The Cell/Tissue Total RNA Kit (Yishan Biotech, Shanghai, China, #RN001) according to the kit instructions. One microgram of RNA was reverse transcribed into cDNA using MonScript RTIII All‐in‐One Mix (Monad, MA, USA; #MR05101). Reverse transcription was performed as follows: reverse transcription for 15 min at 55°C, genomic contamination removal for 2 min at 37°C, and reverse transcriptase inactivation for 5 min at 85°C. Following the acquisition of cDNA, qPCR was carried out via the manufacturer's instructions and MonAmp ChemoHS qPCR Mix (Monad, #MQ00401) on a Bio‐Rad CFX96 Real‐Time PCR instrument. Predenaturation at 95°C for 10 min, denaturation at 95°C for 10 s, and annealing/extension at 60°C for 30 s were used for the qPCR. The latter two steps were performed for 40 cycles. Table S2 displays the primers used for qPCR, with GAPDH serving as the internal reference primer.

### 2.11 Xenograft Tumour Model

Six‐week‐old female BALB/c nude mice were purchased from Shanghai Experimental Animal Center (Shanghai, China). The mice were divided into low, medium, and high weight groups according to body weight, and subsequently one mouse from each of the three groups was randomly selected to form a group for further analysis: the SPARC‐overexpressing negative control (OE‐NC) group (*n* = 3), the SPARC‐overexpressing (OE‐SPARC) group (*n* = 3) and the OE‐SPARC +2‐deoxy‐D‐glucose (2‐DG) group (*n* = 3). In the OE‐NC group, 100 μL of OE‐NC HCT116 cells (7 × 10^6^) were injected subcutaneously into the central flank area of each mouse. The latter two groups were injected with the same number of SPARC‐overexpressing HCT116 cells. 5‐FU (23 mg/kg) was injected into the tail vein of each mouse once every 3 days from the third day, following the manufacturer's instructions for experimental animal use (MCE). The 2‐DG group was intraperitoneally injected with 2‐DG every week (50 mg/kg), while the remaining groups were injected with the same volume of PBS. Tumour size was measured every 3 days starting on the third day in a blind manner, and tumour volume was calculated. Mice were sacrificed via cervical dislocation in accordance with approved ethical guidelines; the tumours were removed and used for IHC experiments.

### 2.12 Statistical Analysis

GraphPad Prism (9.0) was used to perform the statistical analysis of the data. Student's *t* test was used to assess normally distributed data, and the Wilcoxon rank‐sum test was used to analyse nonnormally distributed data. The interaction between SPARC and clinicopathological features was assessed via the chi‐square test. Survival analysis was performed via the Kaplan–Meier method. *p* < 0.05 was considered to indicate statistical significance.

## 3 Results

### 3.1 SPARC Is Highly Expressed in CRC Tissues and Is Associated With a Poor Prognosis in CRC Patients

**FIGURE 1:**
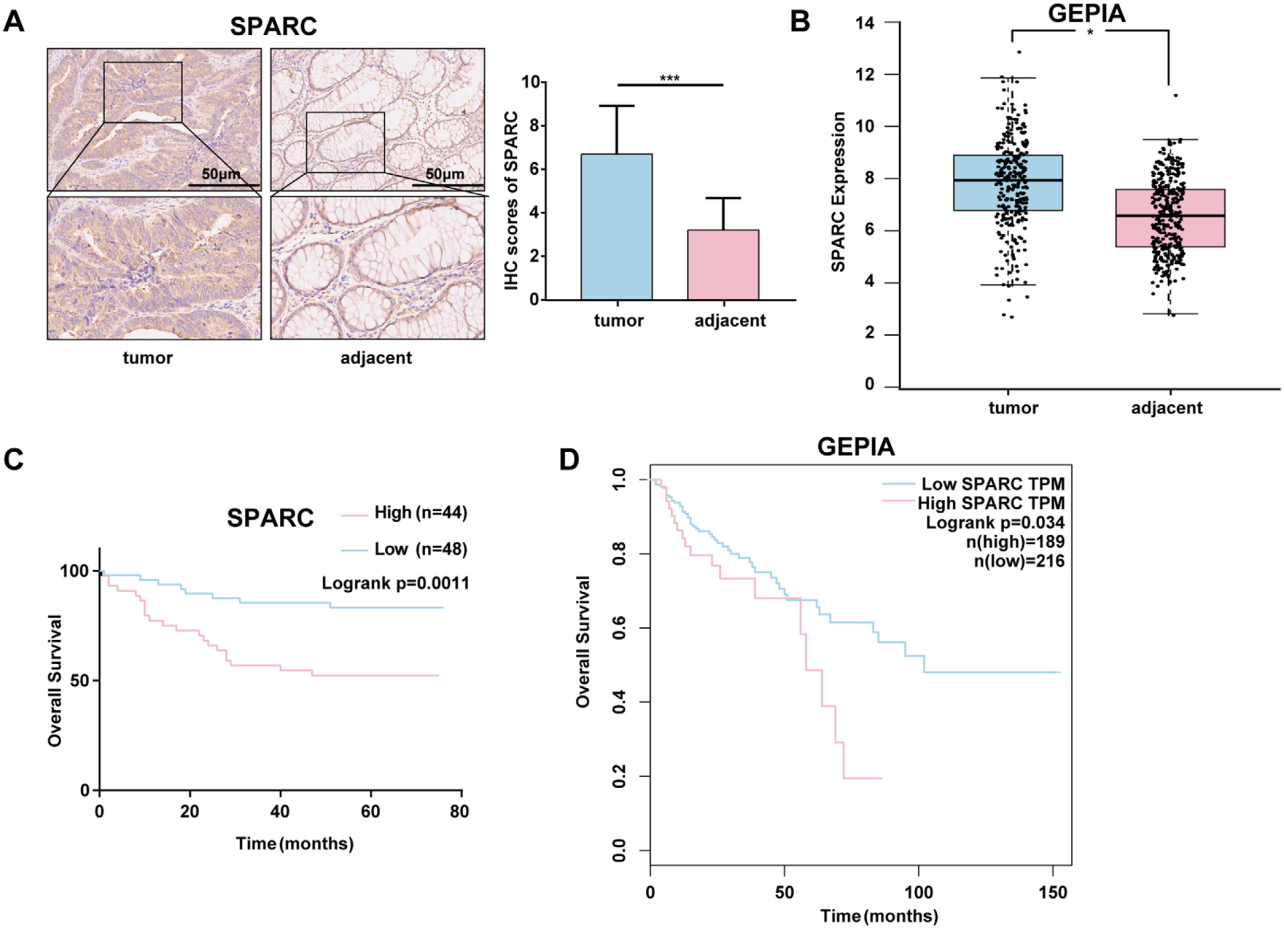
SPARC expression is increased in CRC tissues and is associated with poor prognosis. (A) SPARC expression in CRC tissue microarrays in cancer and adjacent tissues. Scale bar, 50 μm. (B) SPARC expression in cancer and adjacent tissues in the GEPIA database. (C) Kaplan–Meier curves of overall survival for CRC patients in TMAs (log‐rank *p* = 0.0011). (D) Kaplan–Meier curves of overall survival for CRC patients in the GEPIA database (log‐rank *p* = 0.034). Non‐significant results were denoted as “NS” while statistical significance was indicated as *p < 0.05 and ***p < 0.001.

We first determined the expression of SPARC in CRC clinical samples via IHC experiments. Compared with that in adjacent tissues, SPARC expression was considerably increased in CRC tissues (Figure 1A). According to the GEPIA database, SPARC mRNA was strongly elevated in CRC tissue samples (Figure 1B). Furthermore, Table 1 demonstrates a significant association between elevated SPARC expression and advanced nodal metastasis (N stage) as well as higher AJCC staging in CRC patients. More significantly, patients with high expression levels of SPARC had shorter survival times according to Kaplan–Meier survival analysis (Figure 1C,D). The aforementioned findings indicate that SPARC is strongly correlated with a poor prognosis for CRC patients and is highly expressed in CRC tissues.

**TABLE 1:** Clinical features and SPARC expression in patient samples with CRC.

Characteristic	SPARC expression		*p*
	Low	High	
Sex			
Male	26	19	0.292
Female	22	25	
Age			
< 60	13	19	0.105
> 60	35	25	
T			
T1‐2	5	4	1.000
T3‐4	43	40	
N			
N0	35	22	0.024*
N1‐3	13	22	
M			
M0	47	40	0.189
M1	1	4	
AJCC stage			
I‐II	34	21	0.024*
III‐IV	14	23	

* *p* < 0.05.

### 3.2 SPARC Promotes 5‐FU Resistance in CRC

**FIGURE 2:**
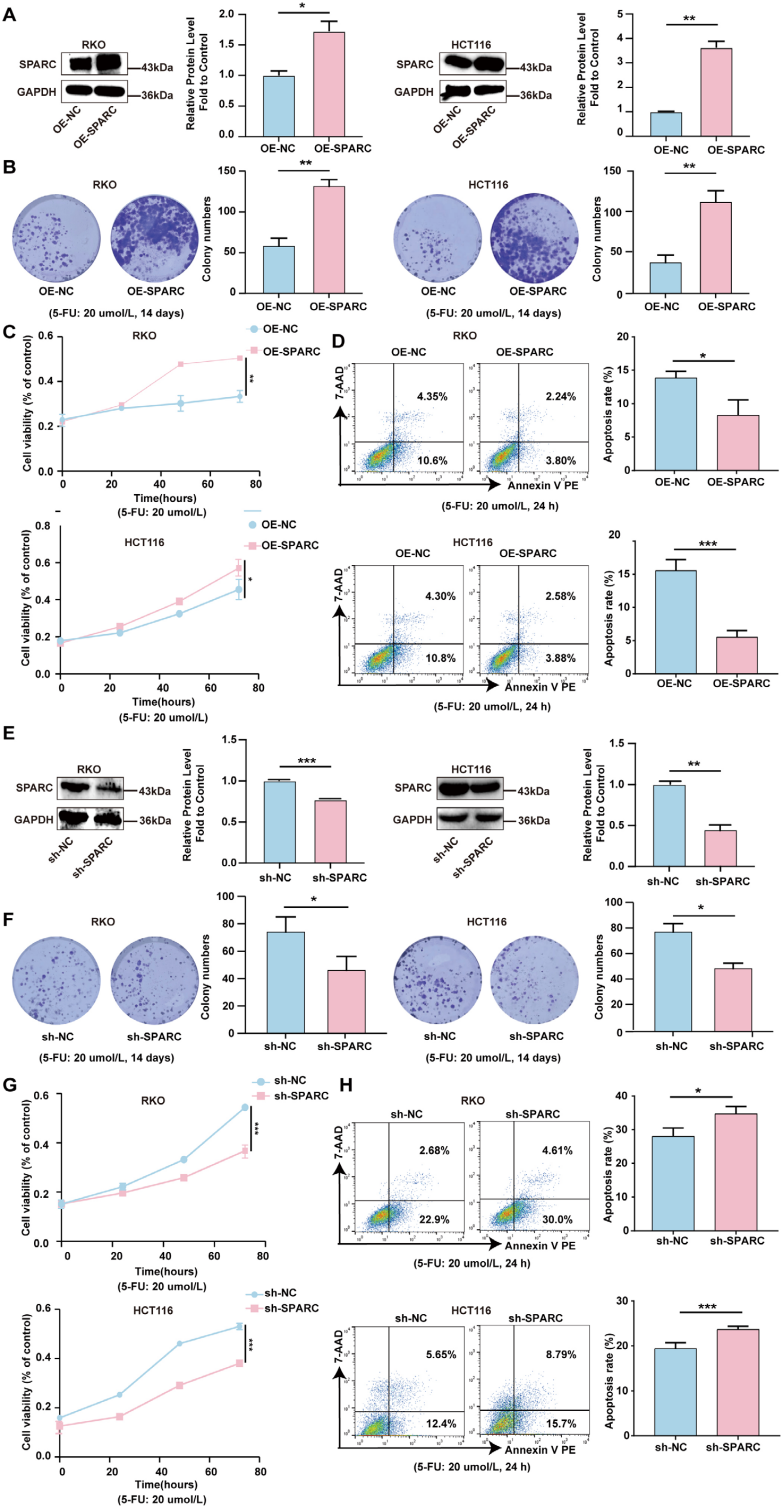
SPARC promotes chemotherapy resistance to 5‐FU in CRC. (A) The protein expression of SPARC in RKO and HCT116 cells transfected with SPARC overexpression lentivirus. (B) Colony formation assays were used to measure the proliferation ability of OE‐SPARC RKO and HCT116 cells compared with OE‐NC cells (5‐FU: 20 umol/L). (C) CCK8 assays were used to detect the cell viability of OE‐SPARC RKO and HCT116 cells compared with OE‐NC cells (5‐FU: 20 umol/L). (D) Flow cytometry was used to determine the apoptosis rate of OE‐SPARC RKO and HCT116 cells compared with OE‐NC cells (5‐FU: 20 umol/L). (E) The protein expression of SPARC in RKO and HCT116 cells transfected with SPARC knockdown lentivirus. (F) Colony formation assays were used to measure the proliferation ability of sh‐SPARC RKO and HCT116 cells compared with sh‐NC cells (5‐FU: 20 umol/L). (G) CCK8 assays were used to detect the cell viability of sh‐SPARC RKO and HCT116 cells compared with sh‐NC cells (5‐FU: 20 umol/L). (H) Flow cytometry was used to determine the apoptosis rate of sh‐SPARC RKO and HCT116 cells compared with sh‐NC cells (5‐FU: 20 umol/L). Non‐significant results were denoted as “NS” while statistical significance was indicated as *p < 0.05, **p < 0.01, and ***p < 0.001.

To investigate the possible biological role of SPARC in CRC, stable SPARC‐overexpressing (OE‐SPARC) RKO and HCT116 cell lines were established. Compared with that in the negative control (OE‐NC) cells, the protein expression of SPARC was significantly elevated in the SPARC‐overexpressing RKO and HCT116 cells (Figure 2A). As shown in Figure 2B, the colony formation assay results revealed that the number of colonies formed by SPARC‐overexpressing CRC cells was greater than that of control cells after 5‐FU treatment (20 μmol/L). Similarly, the results of the CCK‐8 experiments revealed that the overexpression of SPARC promoted the proliferation of CRC cells under 5‐FU treatment (Figure 2C). Moreover, the apoptosis rate of SPARC‐overexpressing RKO and HCT116 cells was significantly lower than that of the control cells under 5‐FU treatment (Figure 2D).

Next, we established stable SPARC‐knockdown RKO and HCT116 cell lines to further explore the effect of SPARC on 5‐FU resistance in CRC (Figure 2E). Colony formation and CCK‐8 assay revealed that SPARC knockdown significantly reduced the survival rate of RKO and HCT116 cells. Furthermore, the flow cytometry‐based apoptosis assay results revealed that depletion of SPARC markedly increased the rate of apoptotic cells among CRC cells under 5‐FU treatment (Figure 2H).

### 3.3 SPARC Promotes Aerobic Glycolysis in CRC Cells via HK2

**FIGURE 3:**
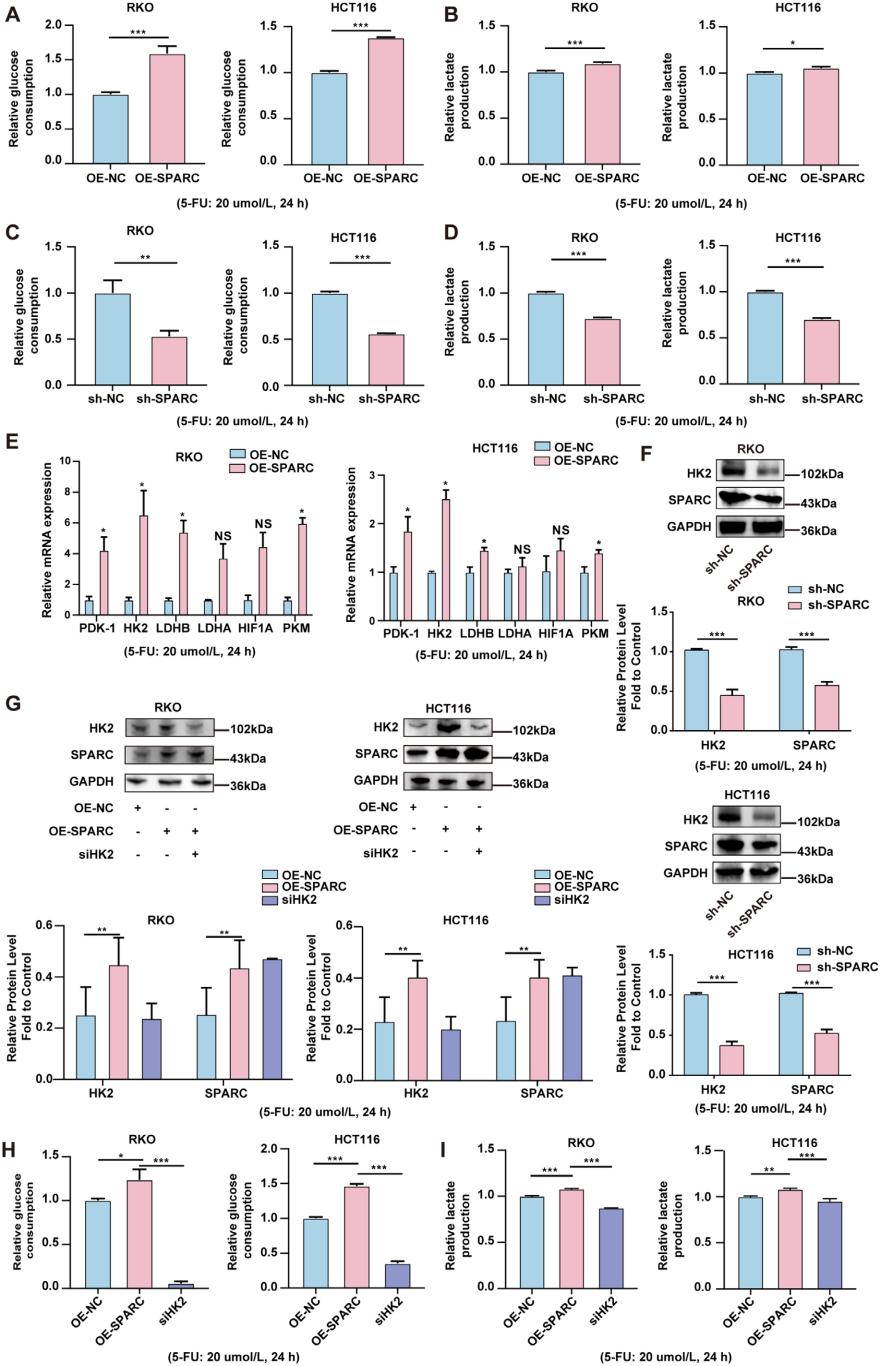
SPARC promotes aerobic glycolysis via HK2 in CRC. (A) Glucose consumption was measured in OE‐SPARC RKO and HCT116 cells compared with OE‐NC cells (5‐FU: 20 umol/L). (B) Lactate production was measured in OE‐SPARC RKO and HCT116 cells compared with OE‐NC cells. (5‐FU: 20 umol/L) (C) Glucose consumption was measured in sh‐SPARC RKO and HCT116 cells compared with sh‐NC cells (5‐FU: 20 umol/L). (D) Lactate production was measured in sh‐SPARC RKO and HCT116 cells compared with sh‐NC cells (5‐FU: 20 umol/L). (E) RT‐qPCR assays were used to analyze the mRNA expression of key genes in aerobic glycolysis in OE‐SPARC RKO and HCT116 cells compared with OE‐NC cells (5‐FU: 20 umol/L). (F) The protein expression of SPARC and HK2 in sh‐SPARC RKO and HCT116 cells was compared with sh‐NC cells (5‐FU: 20 umol/L). (G) The protein expression of SPARC and HK2 in OE‐SPARC RKO and HCT116 cells after treatment with HK2 siRNA (5‐FU: 20 umol/L). (H) Glucose consumption was measured in OE‐SPARC RKO and HCT116 cells after treatment with HK2 siRNA (5‐FU: 20 umol/L). (I) Lactate production was measured in OE‐SPARC RKO and HCT116 cells after treatment with HK2 siRNA (5‐FU: 20 umol/L). Non‐significant results were denoted as “NS” while statistical significance was indicated as *p < 0.05, **p < 0.01, and ***p < 0.001.

Previous studies have shown that aerobic glycolysis is closely associated with the chemoresistance of cancer cells [20]. In addition, SPARC has been reported to be an important regulator of aerobic glycolysis [21, 22]. Hence, we hypothesised that SPARC might regulate 5‐FU resistance in CRC cells through the modulation of aerobic glycolysis. To test this hypothesis, we measured glucose consumption and lactate production in CRC cells. As shown in Figure 3A,B, SPARC overexpression significantly increased glucose consumption and lactate production in CRC cells. In contrast, SPARC knockdown significantly reduced glucose consumption and lactate production (Figure 3C,D). Notably, SPARC overexpression markedly upregulated the expression of aerobic glycolysis‐related genes, including PDK‐1, HK2, LDHB, LDHA, HIF1A, and PKM, in CRC cells (Figure 3E). Among these genes, HK2 exhibited the most significant elevation in mRNA expression with SPARC expression in RKO and HCT116 cells. As expected, at the protein level, SPARC knockdown led to the inhibition of HK2 expression, while SPARC overexpression resulted in the promotion of HK2 protein expression in CRC cells (Figure 3F,G). We subsequently used a commercial HK2 siRNA to investigate whether HK2 was involved in SPARC‐mediated regulation of aerobic glycolysis in CRC. Transfection with HK2 siRNA reversed the SPARC‐mediated increase in HK2 expression in CRC cells (Figure 3G). Furthermore, we found that glucose consumption and lactate production, which were originally elevated by SPARC overexpression, were significantly reduced by HK2 knockdown (Figure 3H,I). These results suggest that SPARC increases aerobic glycolysis in CRC via HK2.

### 3.4 SPARC Promotes Resistance to 5‐FU in CRC via HK2

**FIGURE 4:**
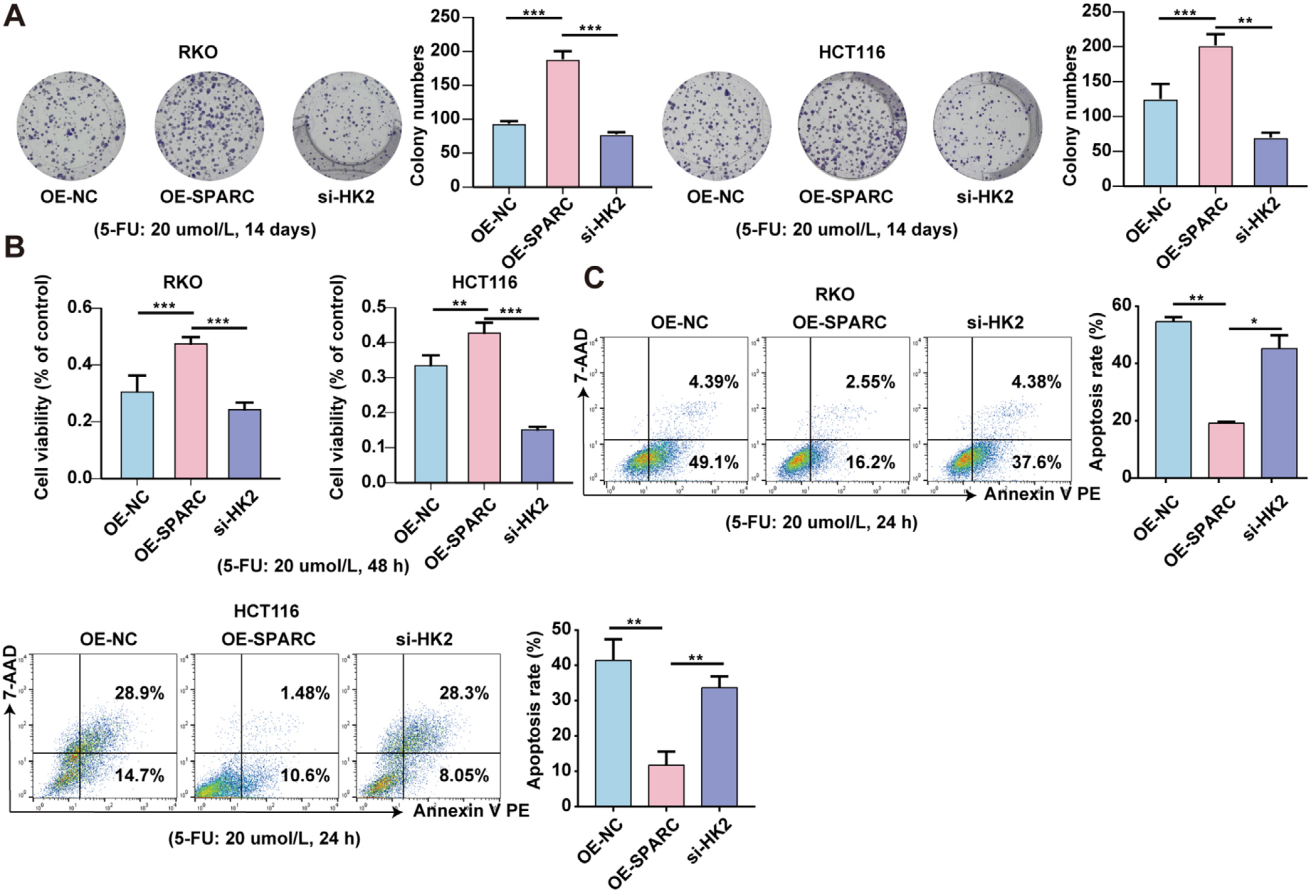
SPARC promotes resistance to 5‐FU chemotherapy as mediated by HK2 in CRC. (A) Colony formation assays were used to measure the proliferation ability of OE‐SPARC RKO and HCT116 cells after being treated with HK2 siRNA (5‐FU: 20 umol/L). (B) CCK8 assays were used to detect the cell viability of OE‐SPARC RKO and HCT116 cells after being treated with HK2 siRNA (5‐FU: 20 umol/L). (C) Flow cytometry was used to determine the apoptosis rate of OE‐SPARC RKO and HCT116 cells after being treated with HK2 siRNA (5‐FU: 20 umol/L). Non‐significant results were denoted as “NS” while statistical significance was indicated as *p < 0.05, **p < 0.01, and ***p < 0.001.

To investigate whether HK2 is involved in the SPARC‐mediated 5‐FU chemoresistance in CRC, we performed colony formation and CCK‐8 assays and found that the knockdown of HK2 abolished the effects of SPARC overexpression on the proliferative capacity and viability of RKO and HCT116 cells under 5‐FU treatment (Figure 4A,B). Additionally, the reduction in the apoptosis rate induced by SPARC overexpression was significantly increased with the knockdown of HK2 (Figure 4C).

### 3.5 SPARC Promotes Resistance to 5‐FU Chemotherapy in CRC by Activating the STAT3/HK2 Axis

**FIGURE 5:**
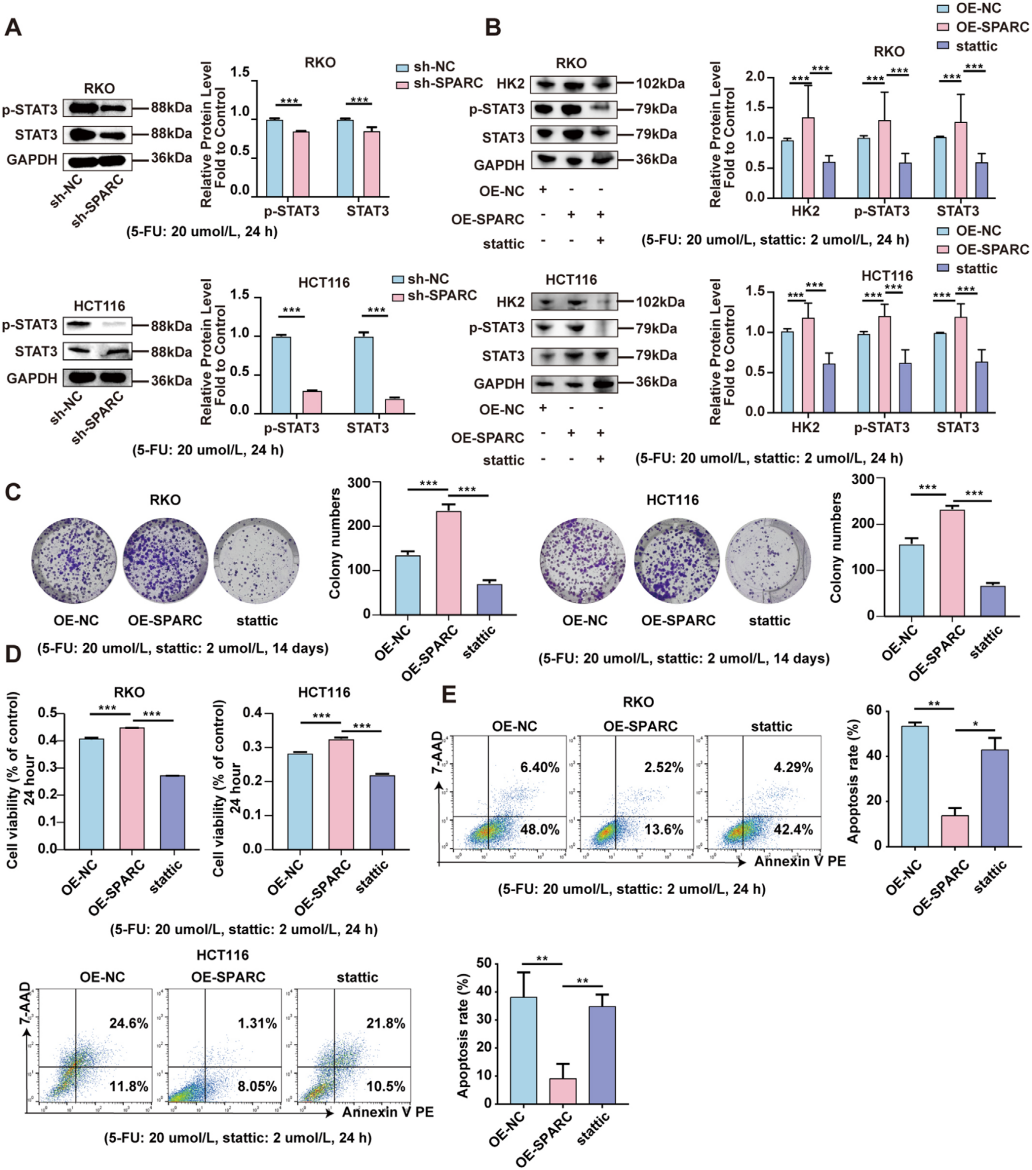
SPARC promotes resistance to 5‐FU chemotherapy as mediated by the STAT3/HK2 axis in CRC. (A) The protein expression of P‐STAT3 and STAT3 in sh‐SPARC RKO and HCT116 cells compared with sh‐NC cells (5‐FU: 20 umol/L). (B) The protein expression of HK2, P‐STAT3, and STAT3 in OE‐SPARC RKO and HCT116 cells after being treated with HK2 siRNA (5‐FU: 20 umol/L, stattic: 2 μmol/L). (C) Colony formation assays were used to measure the proliferation ability of OE‐SPARC RKO and HCT116 cells after being treated with stattic (5‐FU: 20 umol/L, stattic: 2 μmol/L). (D) CCK8 assays were used to detect the cell viability of OE‐SPARC RKO and HCT116 cells after being treated with stattic (5‐FU: 20 umol/L, stattic: 2 μmol/L). (E) Flow cytometry was used to determine the apoptosis rate of OE‐SPARC RKO and HCT116 cells after treated with stattic (5‐FU: 20 umol/L, stattic: 2 μmol/L). Non‐significant results were denoted as “NS” while statistical significance was indicated as *p < 0.05, **p < 0.01, and ***p < 0.001.

In our previous study, we reported that activation of the STAT3 pathway increases HK2 expression in CRC cells [23]. Given that the expression of SPARC is strongly correlated with the STAT3 signalling pathway [24, 25], we wondered whether SPARC‐mediated upregulation of HK2 in CRC cells is associated with the STAT3 signalling pathway. As shown in Figure 5A,B, SPARC knockdown decreased, whereas SPARC overexpression increased the expression of P‐STAT3 in RKO and HCT116 cells. Importantly, following the manufacturer's instructions (MCE), treatment with stattic (2 μmol/L), a STAT3 inhibitor, markedly abolished the ability of SPARC overexpression to promote HK2 expression in CRC cells (Figure 5B). Furthermore, stattic reversed the effects of SPARC overexpression on 5‐FU resistance in CRC cells, as evidenced by the colony formation, CCK‐8, and flow cytometry‐based apoptosis assays (Figure 5C–E). These results suggested that SPARC can upregulate the expression of HK2 and 5‐FU resistance via the STAT3 signalling pathway in CRC cells.

### 3.6 The SPARC/STAT3/HK2 Axis Promotes 5‐FU Resistance in CRC Cells In Vivo

**FIGURE 6:**
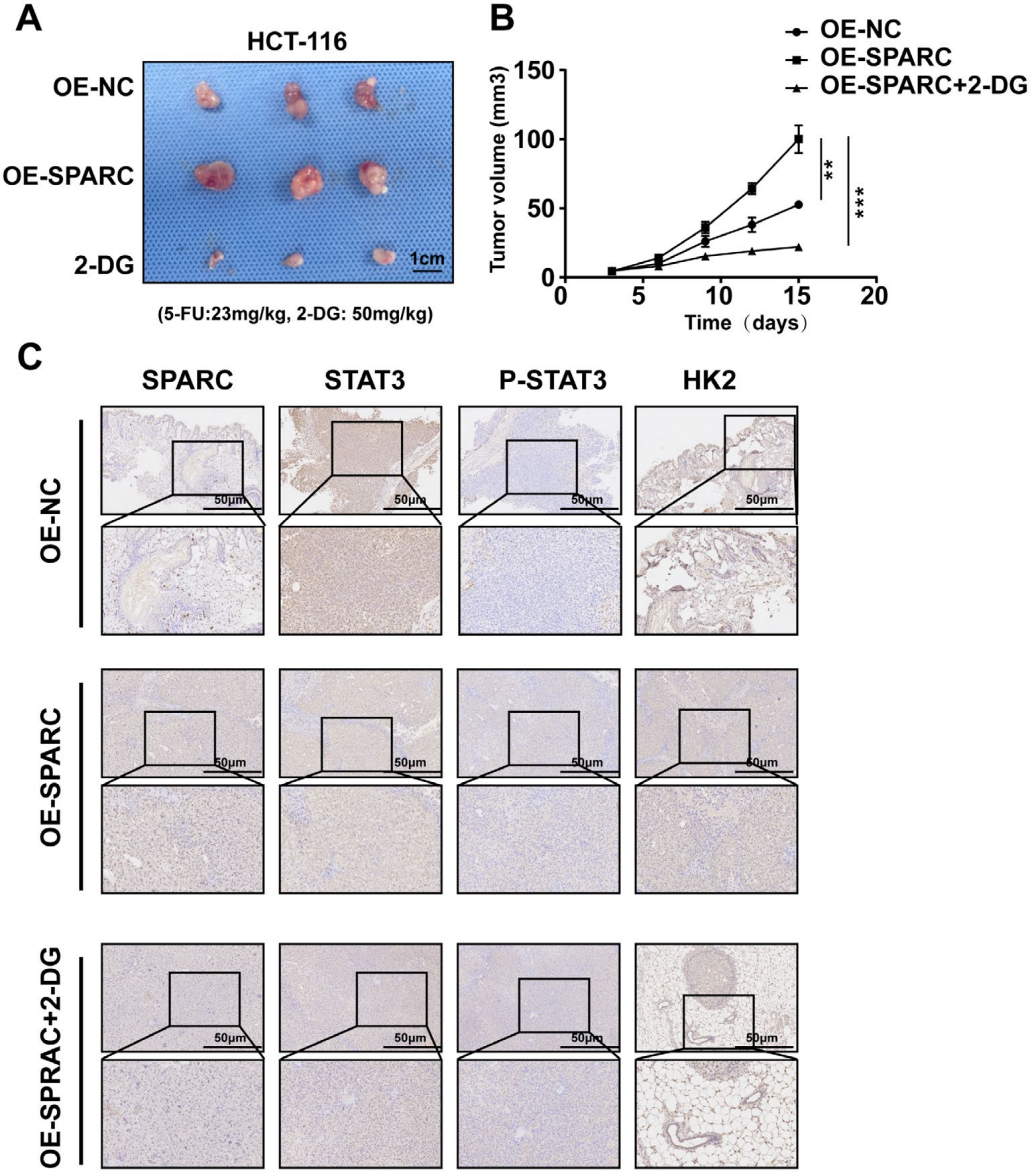
The SPARC/STAT3/HK2 axis promoted resistance to 5‐FU chemotherapy of CRC cells in vivo. (A) Representative images of subcutaneous tumour tissue were acquired from the OE‐NC, OE‐SPARC, and 2‐DG mouse models (5‐FU:23 mg/kg, 2‐DG: 50 mg/kg). (B) The volume and growth rate of subcutaneous tumours in the OE‐NC, OE‐SPARC, and 2‐DG groups were calculated. (C) The expression of SPARC, STAT3, P‐STAT3, and HK2 in subcutaneous tumour tissues in the OE‐NC, OE‐SPARC, and 2‐DG groups were determined by IHC experiments. Scale bar, 50 μm. Non‐significant results were denoted as “NS” while statistical significance was indicated as **p < 0.01 and ***p < 0.001.

To investigate whether SPARC plays a role in promoting resistance to 5‐FU in CRC cells in vivo, xenograft models were established. As shown in Figure 6A,B, under 5‐FU treatment, the tumour volume of the mice in the SPARC overexpression group was significantly larger than that in the control group, and this phenomenon was inhibited by the HK2 inhibitor 2‐DG. We subsequently performed IHC experiments on mouse tumour tissues and found that the intensity of SPARC, P‐STAT3, and HK2 staining was significantly greater in tumour tissues from the OE‐SPARC group than in those from the control group (Figure 6C). However, the administration of 2‐DG decreased HK2 expression but had no influence on SPARC or P‐STAT3 expression (Figure 6C).

## 4 Discussion

Chemotherapy has long been considered an essential part of tumor therapy [26]. For CRC, 5‐FU is the oldest and most important chemotherapeutic agent [27]. Nevertheless, an overwhelming majority of CRC patients are resistant to 5‐FU, which considerably decreases the effectiveness of the remedy [5]. Therefore, understanding the mechanisms by which CRC develops 5‐FU resistance is particularly important. SPARC, as a secreted protein, is thought to regulate the interaction between cells and the extracellular matrix [28] and has powerful biological functions, especially in pathological conditions such as cancer. Recently, the role of SPARC in cancer chemoresistance has received increasing attention. In gastric cancer, the recombinant SPARC protein markedly influenced 5‐FU chemosensitivity in gastric cancer cells via the modulation of epithelial–mesenchymal transition and apoptosis [15]. Yuan et al. reported that SPARC was associated with SOX8‐mediated chemoresistance to albumin‐bound paclitaxel in pancreatic cancer [16]. The link between Caspase‐8 and Bcl‐2 may be disrupted by the SPARC peptide, which can restore sensitivity in chemoresistant cancers [17]. Our research revealed that SPARC functions as a critically important intracellular protein, rather than serving primarily as an exocrine protein, is significantly overexpressed in CRC tissues and is linked to a poor prognosis in CRC patients. Additionally, in vitro and in vivo, SPARC knockdown significantly suppressed aerobic glycolysis and attenuated 5‐FU resistance in CRC cells, whereas SPARC overexpression was increased. Thus, we deduced that in CRC, aerobic glycolysis and resistance to 5‐FU may be significantly influenced by SPARC.

Glycolysis refers to the propensity of cancer cells, especially under normoxic conditions, to metabolise glucose anaerobically rather than aerobically [29]. There is increasing evidence that glycolysis contributes to chemoresistance in a variety of cancers, including CRC [30]. For example, in CRC, HIF‐1α causes glucose metabolic reprogramming and confers 5‐FU resistance by activating the Wnt/β‐catenin and ROS/PI3K/Akt pathways [31]. The long noncoding RNA LINC01852 suppressed tumorigenesis and chemoresistance in CRC by inhibiting PKM2‐mediated glycolysis [32]. Our results showed that SPARC increased aerobic glycolysis in CRC in an HK2‐dependent manner. HK2, a member of the hexokinase family, is widely expressed in tissues but at lower levels than its family member, HK1. HK2 is abundantly expressed in insulin‐sensitive regions such as cardiac, skeletal muscle, and adipose tissues [33], and its expression is also markedly elevated in highly oxygen‐consuming tissues, such as tumour tissues [34]. As a key enzyme in glucose metabolism, it plays an important role in hypermetabolic tumour cells. Tao Zhang et al. (2019) demonstrated the therapeutic effect of targeting the ROS/PI3K/AKT/HIF‐1α/HK2 axis in breast cancer [35]. In addition, the circadian rhythm of the PER1/HK2 axis was shown to be significantly correlated with trastuzumab resistance in gastric cancer [36]. Importantly, both in vitro and in vivo, HK2 knockdown or inhibition eliminated the effects of SPARC overexpression on glycolysis in aerobic environments and 5‐FU resistance in CRC. These findings imply that SPARC controls HK2‐mediated glycolysis to increase 5‐FU resistance in CRC. Hua et al. reported that, in hepatocellular carcinoma, the overexpression of SPARC inhibits glycolysis, resensitizing 5‐FU‐resistant cells to 5‐FU [37]. We hypothesised that the diverse roles of SPARC in a range of cancers and its importance in cancer are contingent upon the specific type of tumour.

The STAT3 signalling pathway, as a collection of multiple oncogenic signalling pathways, plays powerful and diverse roles in a wide range of diseases and tumours. STAT3 has been reported to be involved in the development of chemoresistance in multiple cancers [38, 39]. The inhibition of the JAK/STAT3 axis blocks breast cancer stem cell self‐renewal and fatty acid β‐oxidation, leading to resensitization to chemotherapy [40]. Our earlier findings revealed that B7‐H3 increased CRC chemoresistance via the STAT3/HK2 pathway [23]. Furthermore, a substantial correlation between SPARC and the STAT3 signalling pathway has been shown [24, 25]. Thus, we postulated that SPARC influences 5‐FU resistance and HK2 expression in CRC cells via the STAT3 signalling pathway. As anticipated, the effects of SPARC on HK2 expression and 5‐FU susceptibility in CRC cells were reversed via treatment with stattic, a STAT3 inhibitor. Our results suggested that SPARC promoted 5‐FU resistance and HK2 expression in CRC by increasing the activity of the STAT3 pathway. While our previous study demonstrated the role of B7‐H3 in glycolysis and chemoresistance, the current study expands this understanding by linking SPARC to both HK2 expression and glycolysis regulation. This dual role of SPARC in metabolic reprogramming—specifically through HK2‐mediated glycolysis—adds a new dimension to the mechanisms underlying CRC chemoresistance. Moreover, the potential interplay between B7‐H3 and SPARC in CRC resistance warrants further investigation, such as whether these pathways act synergistically or independently. This comparison highlights the unique contributions of SPARC and positions the study as a complementary advancement in the field.

Finally, this study has several notable limitations. For instance, the mechanism by which SPARC promotes STAT3 phosphorylation remains unclear, and the influence of STAT3 phosphorylation on HK2 requires further investigation. In addition, the primary objective of this study is to investigate the intracellular tumour‐promoting function of SPARC, particularly its role in mediating 5‐FU chemotherapy resistance. SPARC, as an exocrine protein, is bound to play a more important role in the tumour microenvironment, which is worthy of further exploration.

## 5 Conclusion

**FIGURE 7:**
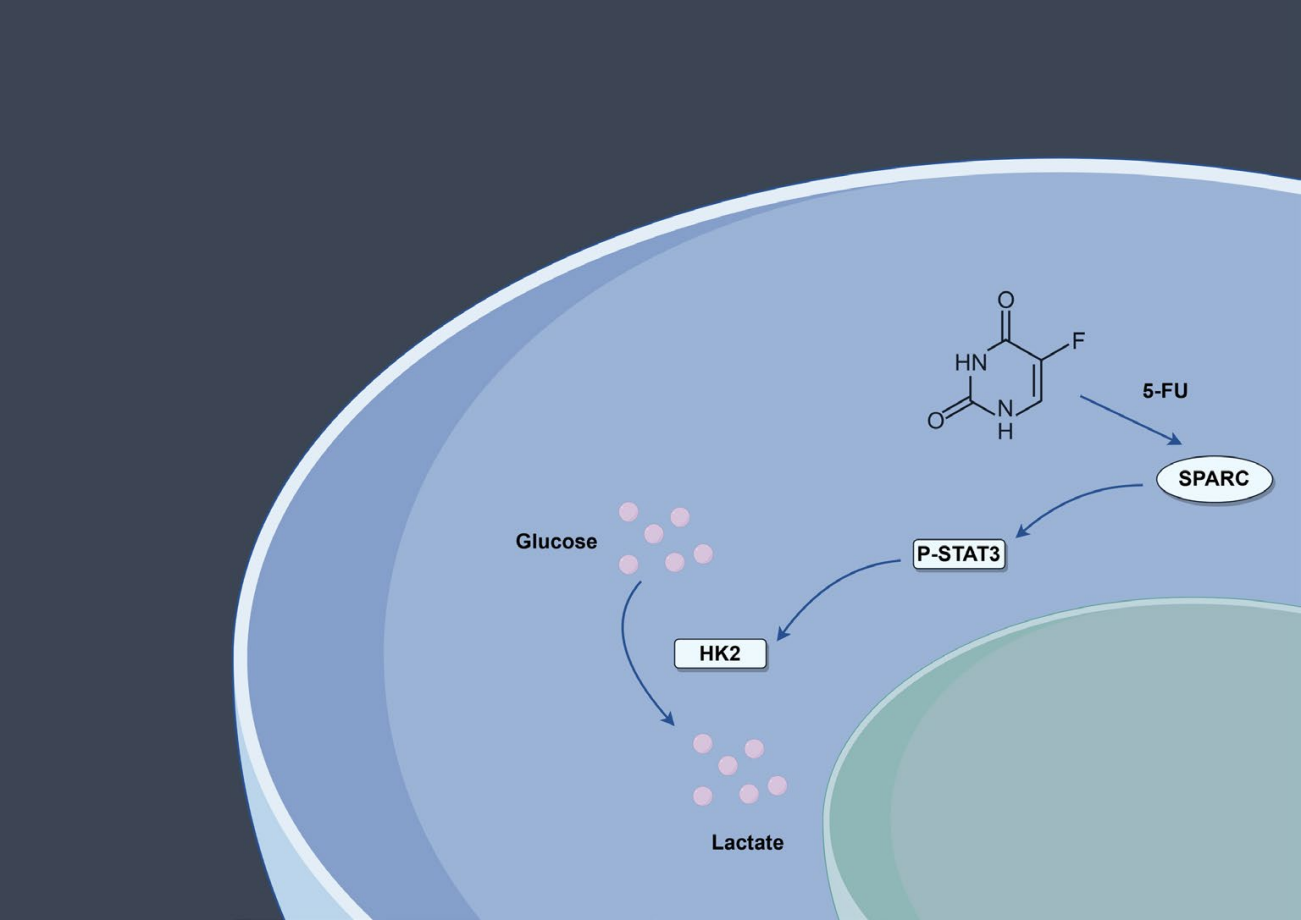
Schematic representation of the mechanism by which SPARC promotes aerobic glycolysis and 5‐fluorouracil resistance in colorectal cancer through the STAT3/HK2 axis.

Overall, our study demonstrated the unique role of SPARC in aerobic glycolysis and resistance to 5‐FU chemotherapy in CRC and successfully illustrated the important impact of the SPARC/STAT3/HK2 axis in this process (Figure 7). A targeted therapeutic strategy that inhibits SPARC to attenuate resistance to 5‐FU chemotherapy in CRC patients may be a promising therapeutic approach for CRC.

## Author Contributions

**Jingrong Xiang:** writing – review and editing (equal), writing – original draft (equal), project administration (equal), data curation (equal). **Huan Zhang:** visualization (equal), software (equal). **Kanger Shen:** writing – review and editing (equal), visualization (equal), supervision (equal), resources (equal), investigation (equal). **Kexi Yang:** formal analysis (equal), methodology (equal). **Jie Feng:** methodology (equal). **Tongguo Shi:** visualization (equal), investigation (equal). **Qinhua Xi:** project administration (equal), funding acquisition (equal).

## Ethics Statement

The experiments involving clinical samples were approved by the Institutional Review Board of the First Affiliated Hospital of Soochow University (No. 2024508). For animal experiments, all of the experimental procedures were performed in accordance with protocols approved by the Institutional Animal Care and Use Committee of Soochow University (Suzhou, China) (No. 202409A0692).

## Conflicts of Interest

The authors declare no conflicts of interest.

## Supporting information


**Table S1:** Antibodies for western bolting.

**Table S2:** Primers for qRT‐PCR assay of gene.

**Table S3:** Characteristics of CRC patients.

## Data Availability

The original contributions presented in the study are included in the article/Supporting Information. Further inquiries can be directed to the corresponding author.
