# Correction to “SPARC Promotes Aerobic Glycolysis and 5‐Fluorouracil Resistance in Colorectal Cancer Through the STAT3/HK2 Axis”

**DOI:** 10.1002/cam4.70998

**Published:** 2025-07-01

Xiang J, et al. SPARC Promotes Aerobic Glycolysis and 5‐Fluorouracil Resistance in Colorectal Cancer Through the STAT3/HK2 Axis. Cancer Med. 2025 Jun;14(11):e70972.

In Figure 2D, an error occurred during the preparation of the figure. The error is that incorrect representative flow cytometric plots were inadvertently selected. This has now been corrected and is shown below. The updated Figure 2D includes the correct representative plots, which accurately reflect the results of our experiment. This error does not affect the overall conclusions of the study.

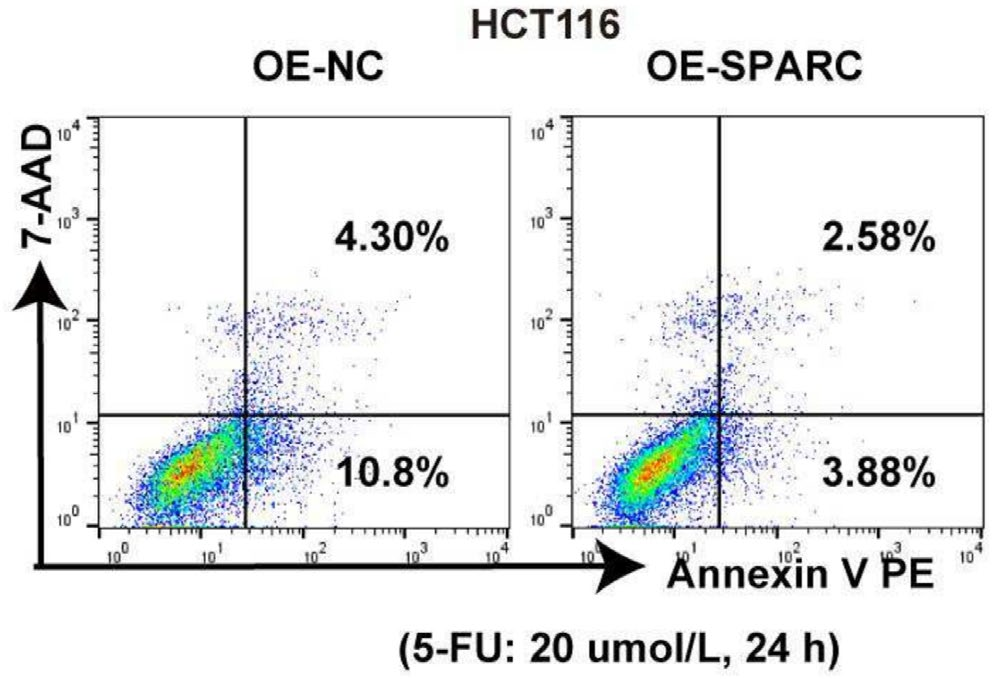

We apologize for this error.

